# Time-Resolved Fluorescence Immunochromatography Assay (TRFICA) for Aflatoxin: Aiming at Increasing Strip Method Sensitivity

**DOI:** 10.3389/fmicb.2020.00676

**Published:** 2020-05-06

**Authors:** Hui Li, Du Wang, Xiaoqian Tang, Wen Zhang, Qi Zhang, Peiwu Li

**Affiliations:** ^1^Oil Crops Research Institute, Chinese Academy of Agricultural Sciences, Wuhan, China; ^2^Key Laboratory of Biology and Genetic Improvement of Oil Crops, Ministry of Agriculture, Wuhan, China; ^3^Laboratory of Quality & Safety Risk Assessment for Oilseed Products (Wuhan), Ministry of Agriculture, Wuhan, China; ^4^Key Laboratory of Detection for Mycotoxins, Ministry of Agriculture, Wuhan, China; ^5^Quality Inspection & Test Center for Oilseed Products, Ministry of Agriculture, Wuhan, China

**Keywords:** time-resolved fluorescence immunochromatography assay (TRFICA), sensitivity, aflatoxin, nanobody, application

## Abstract

Aflatoxin is the most harmful mycotoxin that is ubiquitous in foods and agro-products. Because of its high toxicity, maximum admissible levels of aflatoxins (AF) is regulated worldwide, and monitoring of their occurrence in several commodities is mandatory for assuring food safety and consumers’ health. Considering that the strip method is very simple and convenient for users, in order to enhance strip assay’s sensitivity, a lot of time-resolved fluorescence immunochromatography assays (TRFICAs) were developed recently with increasing several times of assay sensitivity compared with traditional gold nanoparticle-based strip assay (GNP-SA). This review briefly describes the newly developed TRFICA for aflatoxin determination, including TRFICA for aflatoxin B_1_ (AFB_1_) detection, TRFICA for aflatoxin M_1_ (AFM_1_) detection, TRFICA for total aflatoxins (AFB_1_ + B_2_ + G_1_ + G_2_) detection and the latest identification-nanobody-based TRFICA for aflatoxin detection. The application of TRFICA for aflatoxin detection in different agro-products is also concluded in this review. Reasonably, TRFICA has been becoming one of the most important tool for monitoring aflatoxin in foods and agro-products.

## Introduction

Aflatoxin, a group of highly toxic second metabolites from some *Aspergillus* species such as *Aspergillus flavus* and *Aspergillus paraticus*, has still been threatening human health worldwide and the sustainable and high-quality development of modern agriculture industry (Danesh et al., 2018). Aflatoxin caused lots of serious events in the history. In 1960s, aflatoxin was found from the contaminated feeds that caused “turkey event” (Blout, 1961). In 1974, an outbreak of aflatoxin-induced hepatitis occurred in about 200 villages in western India, with maize as the staple food, and in the event, there were 397 patients infected, among which 106 patients died (Krishnamachari et al., 1975; Pitt, 2000). In 2004–2005, Kenya witnessed the largest outbreak of aflatoxin poisoning until now, with more than 1,000 people poisoned and 125 deaths, and the “culprit” was eventually identified as aflatoxin-contaminated maize (Williams et al., 2004; Muture and Ogana, 2005). As reported in China in 2005, the positive rate of aflatoxin M_1_ (AFM_1_) in urine of 300 volunteers from some universities in Guangzhou was as high as 47%. Aflatoxin contamination also resulted in huge economic losses. During the past decade, the ratio of export trade notification from EU caused by aflatoxin exceeded the corresponding government’s maximum limit, close to 30%. Even in developed country like the United States, aflatoxin caused over 225 million dollars loss per year (Robens and Cardwell, 2003).

Sensitive detection method is the key tool for monitoring aflatoxin contamination and preventing contaminated foods far away from table. Aflatoxin, belonging to natural contaminants, was found in many agricultural products (maize, rice, groundnut, etc.), foods (edible plant oil, milk, meat, etc.), and feeds and may occur in the steps of planting field, harvest, transport, storage, process, and even circulation of commodities (Wang B. et al., 2016; Zhao et al., 2018). Therefore, it is most important to find the contamination on time.

As is known, a lot of analytical methods for aflatoxin detection have been developed. Instrumental methods, such as high-performance liquid chromatography (HPLC) with fluorescence detector (Koc et al., 2009; Sheijooni-Fumani et al., 2011; Kong et al., 2013) and ultraperformance liquid chromatography–tandem mass spectrometry (UPLC-MS/MS) (Scholl, 2004; Cervino et al., 2008; Scholl and Groopman, 2008; Deng et al., 2017), may give precise results and especially fit for lab performance but not for on-site detection. However, more and more producers want to use on-site assay methods for monitoring and ensuring their products’ quality. Immunoassays have been developed especially for this aim (Kolosova et al., 2006; Tang et al., 2010; Masinde et al., 2013; Lin et al., 2017; Yao et al., 2017; Pan et al., 2018; Wang et al., 2018; Bhardwaj et al., 2019). Among the developed immunoassays, membrane-based strip assay for aflatoxin determination becomes more and more popular, although it was found to have lower sensitivity than ELISA (Wang et al., 2011; Anfossi et al., 2013). As ELISA is still limited to special equipment in laboratories and is time consuming, these make it not suitable for on-site monitoring (Hu et al., 2017).

Considering that the strip method is very simple and convenient for users, recently, many efforts were made for enhancing strip assay’s sensitivity. Time-resolved fluorescence immunochromatography assay (TRFICA) is one of the important new methods (Rundstrom et al., 2007), which was reported to increase several times of the detection sensitivity compared with that of the traditional gold nanoparticle-based strip assay (GNP-SA). Expensive time-resolved fluorescence detector was ever thought as the bottleneck for TRFICA technology (Liu et al., 2016). During the past 5 years, with the development of portable TRFICA reader, the TRFICA strip has been enhanced quickly and even occurred in the market.

Therefore, we here first made a review for the newly developed analytical technology: TRFICA for aflatoxin detection.

## Principle

TRFICA combines the advantage of time-resolved fluorescence immunoassay (TRFIA) and chromatography, labeling the lanthanide-chelate-embedded nanoparticles or microbeads with antibodies or protein, which are captured in the detection area by the principle of chromatography (Ye et al., 2004; Xia et al., 2009). Lanthanide chelates exhibit highly desirable fluorescence characteristics: (1) long fluorescence lifetime (Eu^3+^ has a lifetime on the order of millisecond, which is several orders of magnitude longer than that of the non-specific background autofluorescence) (Karhunen et al., 2011); (2) wide excitation spectrum and narrow and sharp emission spectrum (Hemmilä, 1995; Matsumoto and Yuan, 2003); and (3) a large Stokes shift (200–300 nm) (Huhtinen et al., 2005; Binnemans, 2009; Ouyang et al., 2011), with improved spatial resolution. Lanthanide chelates were embedded into microbeads to increase fluorescence intensity (Harma et al., 2001; Huhtinen et al., 2004; Kokko et al., 2007), which effectively resolve the limitation of conventional dissociation-enhanced lanthanide fluorescence immunoassay used in liquid phase only (Xia et al., 2013; Xu et al., 2013; Zhang et al., 2014). What is more, through wave resolution and time-delay technique, TRFICA can be used for quantitative detection with high sensitivity, wide linear range, and low background.

**FIGURE 1:**
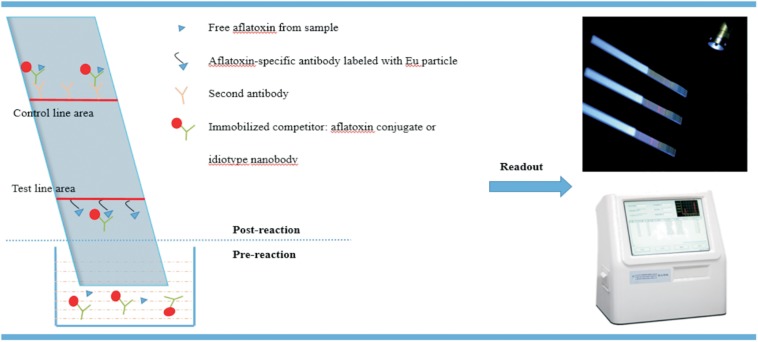
The scheme of time-resolved fluorescence immunochromatography assay (TRFICA) for aflatoxin detection.

A sample pad, nitrocellulose membrane sprayed with test and control lines, and absorbent pad are assembled into a TRFICA device (Tang et al., 2015; Wang et al., 2015; Zhang et al., 2015). The liquid sample in the microreaction pool moves from the sample pad, through nitrocellulose membrane, to the absorbent pad through capillary action. In the absence of aflatoxin, the fluorescent lanthanide microbead-labeled antibodies react with the immobilized antigen (aflatoxin–protein conjugate) on the test line and secondary antibody on the control line, so two colored lines are observed under ultraviolet illumination. However, when aflatoxin presents in the sample, the aflatoxin–protein conjugate immobilized on the test line competes with the aflatoxin in samples to bind with lanthanide microbead-labeled antibodies. Thus, the fluorescence signal in the test zone is negatively correlated with the concentrations of [Frame1]aflatoxin in the sample, as illustrated in Figure 1.

## Development

After the first introduction of Eu^3+^ complexes by Weissman (1942), the TRFICA method was found to be extremely suitable for rapid on-site assay. However, the lack of a portable TRFICA detector greatly limited its wide application in the past few years. In 2015, Wang et al. developed a homemade and portable TRFICA reader for aflatoxin determination. The components of the TRFICA detector are listed as follows: (1) a xenon lamp was used as the excitation source with an excitation wavelength of 365 ± 10 nm; (2) the emission light was collected at 613 ± 10 nm by a photomultiplier tube (PMT) after a 400-μs delay of the excitation light; (3) the interference band-pass filters were used to obtain the specific excitation and emission bands; (4) the emission signals were processed by a rapid preamplifier–discriminator and pulse counter; and (5) the results were delivered to the readout.

### TRFICA for Aflatoxin B_1_

Aflatoxin B_1_ (AFB_1_) is considered as the most toxic and potent naturally occurring carcinogen, which has been classified as a Group 1 human carcinogen (International Agency for Research on Cancer [IARC], 1993; Streit et al., 2012) mainly targeting liver and lungs (Otim et al., 2005; Turner et al., 2009). It can easily contaminate both food and feed during almost every stages, such as before and after harvest and during storage, transportation, and consumption. Consequently, the study on rapid, effective, and on-site determination of AFB_1_ in the food chain has attracted tremendous efforts in the past few years, as indicated in Table 1. Zhang et al. developed a portable TRFICA for the sensitive determination of AFB_1_ (Zhang et al., 2015). First, they fabricated a TRFICA strip. Ovalbumin (OVA) [2% (*w*/*v*)] was used to block the sample pad in order to prevent non-specific adsorption. On the nitrocellulose membrane, the test and control lines were coated with AFB_1_–bovine serum albumin (AFB_1_–BSA) and immunoglobulin G (IgG), respectively. The distance between the test and control lines was set as 5 mm, so an effective detection zone could be obtained. The sample pad, nitrocellulose membrane, and absorbent pad were assembled onto a plastic scale board in turn and then cut into TRFICA strips with a width and length of 4 and 60 mm, respectively. During the analysis process, free AFB_1_ and the monoclonal antibody against AFB_1_ (anti-AFB_1_ mAb)-conjugated fluorescent microbeads (Eu^3+^) were mixed in a reaction pool first and then laterally flowed through the TRFICA strip via capillary action. After specific binding interaction of aflatoxin–antiaflatoxin mAb and antiaflatoxin mAb-IgG, the detection results based on the fluorescence intensity of the test and control lines could be read out in 6 min using the proposed TRFICA detector. TRFICA exhibited a magnified positive signal with low signal/noise ratio due to the absence of background interference fluorescence and scattering light. This platform demonstrated a wider dynamic range from 0.2 to 60 μg/kg, with a lower limit of detection (LOD) from 0.06 to 0.12 μg/kg (12 times diluted sample, be converted to 0.005–0.01 ng/ml). The detection sensitivity was increased by two orders of magnitude than that of the competitive ELISA developed by Kolosova et al. for AFB_1_ detection with an IC_50_ value of 0.62 ng/ml (Kolosova et al., 2006). Compared with the previously developed GNP-SA for AFB_1_ with a visual detection limit (VDL) of 1 ng/ml (Zhang et al., 2011a), the detection sensitivity was also greatly improved.

**TABLE 1:** A list of time-resolved fluorescence immunochromatography assay (TRFICA) for aflatoxin detection in different samples.

Samples	Analyte	LOD (μg/kg)	Linear range (μg/kg)	Recovery (%)	References
Feed	AFB1	0.06	0.2–60	80.5–116.7	Zhang et al., 2015
Soybean sauce	AFB1	0.1	0.3–10.0	87.2–114.3	Wang D. et al., 2016
Maize	AFB1	0.05	0.13–4.54	72.6–106.6	Tang et al., 2017
Chinese herbal medicines	AFB1	0.60	0.60–3.92	73–95%	Sun et al., 2018
Peanut	AFB1	0.18	0.48–20	83.24–110.80	Wang et al., 2019
Milk	AFM1	0.009	0.02–0.4	88.7–105.0%	Li et al., 2018
Feed	AFB1 + B2 + G1 + G2	0.16	0.48–30.0	83.9–113.9	Wang et al., 2015

### TRFICA for AFM_1_

Aflatoxin M_1_, which is converted by AFB_1_ through hydroxylation under liver cytochrome P450 catalysis, is usually excreted from lactating animals that ingest feed contaminated with AFB_1_. It has been classified as a group 2B human carcinogen by the International Agency for Research on Cancer (International Agency for Research on Cancer [IARC], 1993). Aflatoxin M_1_ can be extensively found in milk and dairy products in both developing and developed countries, causing great threat to consumers’ health. Thus, there is an urgent need to develop a rapid and sensitive detection method for AFM_1_ in milk. Tang et al. (2015) developed a highly sensitive TRFICA to detect AFM_1_ in milk. The test line was coated with AFM_1_–BSA conjugate, and the control line was coated with the goat antirabbit IgG on the nitrocellulose membrane. The absorbent pad, nitrocellulose membrane, and the glass fiber sample pad were then assembled and cut into 4 mm × 60 mm TRFICA strips. The anti-AFM_1_ mAb 2C9 exhibited high affinity to AFM_1_ with an affinity constant of 1.74 × 10^9^ l/mol. Under the competitive ELISA format, its IC_50_ reached (50% inhibition concentration of AFM_1_) 0.067 ng/ml, and the cross-reactivity to AFB_1_, B_2_, G_1_, and G_2_ was <0.1%. When anti-AFM_1_ mAb 2C9 was used in this TRFICA with a competitive format, the detection sensitivity for AFM_1_ was 0.03 ng/ml. According to the previously reported GNP-SA, which exhibited a VDL of 0.3 ng/ml for AFM_1_ (Zhang et al., 2012), the detection limit was enhanced an order of magnitude.

### TRFICA for Total Aflatoxins (AFB_1_ + B_2_ + G_1_ + G_2_)

Aflatoxins (AF), including AFB_1_, AFB_2_, AFG_1_, and AFG_2_, can occur in a wide range of commodities, such as cereals, tree nuts, and spices. They cause great concern in the globe due to its heavy threat such as teratogenic, mutation, and cancer to human being health. In order to avoid human exposure to AF, strict limits have been set up by international government agencies. Therefore, it is urgent to develop rapid and sensitive detection methods for AF monitoring in agro-products. Wang et al. (2015) developed a TRFICA for highly sensitive total aflatoxin detection. The AFB_1_-conjugated BSA and rabbit antimouse IgG were coated on the nitrocellulose membrane as a test line and a control line, respectively. The TRFICA strips were made in a width and length of 4.5 and 70 mm. Antiaflatoxin mAb_4__F__12_ was homemade and purified with a protein G immunoaffinity column before use. Its cross-reactivity toward AFB_1_, AFB_2_, AFG_1_, and AFG_2_ were 100, 90.1, 84.6, and 20.7%, respectively. A wide dynamic range from 0.48 to 30.0 μg/kg with a LOD of 0.16 μg/kg (40 times diluted sample, be converted to 0.004 ng/ml) was obtained by this TRFICA for total aflatoxin detection. The detection sensitivity is one order of magnitude higher than that of the GNP-SA for total aflatoxin detection with VDLs from 0.03 to 0.25 ng/ml reported by Zhang et al. (2011b). The detection sensitivity was also higher than that of the ELISA developed by Li et al. for aflatoxin detection with a LOD of 0.06–0.09 ng/ml (Li et al., 2009).

### Idiotype Nanobody-Based TRFICA for Aflatoxin

As is known to all, antibody plays a very important role in immunoassay. In recent years, anti-idiotypic antibodies, also named as anti-idiotypic nanobodies (AIdnbs), which are composed of only heavy chains obtained from camelids, have gained considerable attention due to their unique properties (Muyldermans, 2013; Muyldermans and Lauwereys, 1999; Bazin et al., 2017). They can be prepared in large quantities, and the small size makes them suitable for bioengineering. What is more, the interaction between AIdnbs and its antigen changes from a majority of side-chain contacts to main-chain contact, which makes them useful in molecular mimicry. They also exhibited high solubility and chemical stability compared with traditional mAbs in sample matrix. Combining the unique characteristic of AIdnbs with the advantage of TRFICA, Tang et al. (2017) developed a highly sensitive and green immunoassay for AFB_1_ detection without the use of toxic traditional antigen. For AIdnb-based TRFICA, phages 2–5 (anti-1C11 nanobody) used as capture antigen and rabbit IgG were coated on the nitrocellulose membrane, respectively. During the TRFICA procedure, AFB_1_ in the sample bound to its specific antibody 1C11 first. After the capillary action, the competitive reaction between phages 2–5 and AFB_1_ toward mAb 1C11 occurred on the test line. Under optimal conditions, AIdnb–TRFICA provided a quantitative relationship ranging from 0.13 to 4.54 ng/ml for AFB_1_, with a LOD of 0.05 ng/ml in the buffer solution.

## Main Applications

The TRFICA offers many advantages: (1) simple operation, fast analysis, and less time consuming; (2) practicality for on-site use; (3) high accuracy; and (4) good reproducibility and stability. As the fluorescence lifetime of lanthanide chelates is several orders of magnitude longer than that of the background interference fluorescence in agro-products, it is favorable to collect the fluorescence signal of lanthanide-chelate-embedded microbeads coupled with aflatoxin in the absence of background fluorescence by time delay technique. Therefore, the detection sensitivity and reliability are greatly improved in TRFICA. In recent years, TRFICA has been widely used for aflatoxin detection in several kinds of agro-products.

In 2015, Zhang et al. developed a portable TRFICA for sensitive and on-site determination of AFB_1_ in food and feed samples. This method provided a wide dynamic range of 0.2–60 μg/kg with a LOD from 0.06 to 0.12 μg/kg, and good recovery was also obtained in different food and feed sample matrices from 80.5 to 116.7% (Zhang et al., 2015). A sensitive TRFICA was developed by Wang D. et al. (2016) for the detection of AFB_1_ in soybean sauce with a dynamic range from 0.3 to 10.0 μg/kg and a LOD of 0.1 μg/kg, with recoveries between 87.2 and 114.3%. Tang et al. (2017) developed an AIdnb-based TRFICA for AFB_1_ detection in maize and its products with a dynamic range from 0.13 to 4.54 ng/ml and a LOD of 0.05 ng/ml. Good recoveries (72.6–106.6%) in maize samples were also obtained (Tang et al., 2017). Sun et al. (2018) developed TRFICA for AFB_1_ detection in Chinese herbal medicines. The established TRFICA showed good linear range from 0.60 to 3.92 μg/kg, with a LOD of 0.60 μg/kg in Chinese herbal medicines *Semen coicis*, *Rhizoma dioscoreae*, and *Platycodon grandiflorus*. The average recovery was 73–95% with a relative standard deviation of <9.08% (Sun et al., 2018). Wang et al. (2019) developed a TRFICA for AFB_1_ detection with a linear detection range of 0.48–20 μg/kg and a LOD of 0.18 μg/kg in peanut. The recovery range was 83.24–110.80% (Wang et al., 2019). In 2020, a TRFICA for AFB_1_ detection was established by Chang et al. (2020) with a LOD of 0.04 μg/kg and recoveries ranging from 71.6 to 119.98% in grains.

Li et al. (2018) developed a TRFICA based on a unique bridge-antibody label to realize on-site detection of AFM_1_ in milk. Different from the previous reports of TRFICA, the fluorescent Eu nanoparticles were first conjugated with polyclonal antibodies and then with monoclonal antibodies. According to the detection results, the sensitivity of this newly developed TRFICA was greatly improved compared with monoclonal antibodies-labeled fluorescent Eu nanoparticle-based TRFICA. A linear range from 0.02 to 0.4 ng/ml with a LOD of 0.009 ng/ml was obtained, and the recoveries ranged from 88.7 to 105.0% for AFM_1_.

In 2019, TRFICA was used to detect total AF in corn samples with a calculated limit of quantity of 0.03 ng/ml (Tang et al., 2019). Wang et al. (2015) developed a TRFICA for total aflatoxin detection in feed samples. It showed a wide dynamic range of 0.48–30.0 μg/kg with a LOD of 0.16 μg/kg, and the recoveries ranged from 83.9 to 113.9%. In 397 feed samples from company and livestock farms throughout China, the detection rate of total AF was 78.3%, and the concentrations were in the range of 0.50–145.30 μg/kg (Wang et al., 2015).

## Conclusion

Aflatoxin is the most harmful mycotoxin that is ubiquitous in foods and agricultural supplies. In order to ensure consumption safety, it is very necessary to develop highly sensitive assay methods for aflatoxin detection. GNP-SA is one of the most simple and popular test method. However, its detection sensitivity is usually unsatisfactory for practical use. In order to improve the detection sensitivity, TRFICA has been developed in recent years. TRFICA for AFB_1_, AFM_1_, and total aflatoxin detection in various kinds of agricultural products has been reported. Compared with the previously reported GNP-SA and ELISA, the detection sensitivity was greatly improved (Table 2). Reasonably, TRFICA has been becoming one of the most important tool for monitoring aflatoxin in foods and agro-products. In consideration of the advantages of simple operation and practicality for on-site use, TRFICA can be widely used to detect various food contaminants, such as other biotoxins, pesticide, and so on in the future.

**TABLE 2:** Comparison of the detection sensitivity of time-resolved fluorescence immunochromatography assay (TRFICA) and gold nanoparticle-based strip assay (GNP-SA).

Analyte	LOD (ng/ml)		Compare results (enhanced times)	References
	TRFICA	GNP-SA		
AFB1	0.005	1	Three orders of magnitude	Zhang et al., 2011a, 2015
AFM1	0.03	0.3	One order of magnitude	Zhang et al., 2012; Tang et al., 2015
AF (B1 + B2 + G1 + G2)	0.004	0.03	One order of magnitude	Zhang et al., 2011b; Wang et al., 2015

## Author Contributions

QZ and HL conceived the review and wrote the manuscript. All authors edited, read and approved the final manuscript.

## Conflict of Interest

The authors declare that the research was conducted in the absence of any commercial or financial relationships that could be construed as a potential conflict of interest.
